# Visual Compensation in Online Food Consumption: Linking Visual Perception, Sensory Representation, Taste Expectation, and Purchase Intention

**DOI:** 10.3390/foods15203763

**Published:** 2026-10-21

**Authors:** Chun Yang, Rong Deng, Juncheng Mu, Yue Ding, Xinqiang Chen

**Affiliations:** 1 School of Design, Jiangnan University, Wuxi 214122, China; dr530@jiangnan.edu.cn (R.D.); 6240310007@stu.jiangnan.edu.cn (Y.D.); 2 School of Art, Nanjing University of Information Science and Technology, Nanjing 210044, China; 004120@nuist.edu.cn; 3 School of Economics and Management, Xiamen University of Technology, Xiamen 361024, China; chenxq23@xmut.edu.cn

**Keywords:** online food consumption, visual compensation, taste expectation, purchase intention

## Abstract

The absence of sensory information in online food consumption hinders consumers’ direct taste, olfactory, and tactile experiences prior to purchase. Consequently, visual information becomes a crucial compensatory cue for forming judgments about food sensory attributes. This study proposes an analytical framework centered on visual compensation to investigate how sensory information richness, visual realism, and visual vividness are associated with consumer purchase intention through gustatory mental imagery, texture mental simulation, and taste expectation. Utilizing survey data from 1226 online food consumers, this research employs PLS-SEM, IPMA, and fsQCA for empirical testing. The results indicate that sensory information richness is positively associated with consumers’ perception of visual realism and vividness, which in turn are positively associated with gustatory mental imagery and texture mental simulation. Gustatory mental imagery and texture mental simulation are subsequently associated with taste expectation, while texture mental simulation is also directly associated with purchase intention. IPMA further reveals that taste expectation, characterized by high importance yet relatively low performance, represents a potential priority for improvement, whereas texture mental simulation demonstrates both high importance and high performance. fsQCA findings illustrate that high PI can be achieved through various combinations of visual perception and psychological processing conditions. Moreover, the configurations associated with High PI differ from those associated with Not High PI. This study develops an integrative account of how visual perception, internal sensory representations, and taste expectations are linked to purchase judgments in online food consumption. It also expands the understanding of visual compensation mechanisms in digital food consumption from both structural and configurational perspectives.

## 1. Introduction

The proliferation of digital platforms, mobile devices, and social media has fundamentally altered how consumers acquire food information and make purchasing decisions [1]. Unlike offline consumption, online food purchasing situates products and consumers in spatially separated environments, thereby limiting the multisensory engagement typical of traditional consumption scenarios [2]. Consumers are generally unable to directly taste, smell, or touch food before purchase, rendering online food selection inherently characterized by a deficit in sensory information [3]. This necessitates a greater reliance on visual representations, such as food imagery, to form judgments regarding freshness, palatability, and other potential sensory qualities [4]. Within this context, visual information transcends mere presentation of external appearance and emerges as a vital cue for consumers to form cross-modal expectations. Existing literature indicates that visual features like color and shape can influence consumer expectations and perceptions of taste and flavor [5]. Furthermore, digital visual representations can prompt consumers to mentally simulate the eating process and its outcomes in the absence of actual consumption experience, thereby impacting their purchasing judgments [6]. Consequently, the capacity of visual information to translate from external perceptual cues into internal representations of food’s sensory attributes and potential consumption experiences is a critical determinant of online food consumption decisions.

Visual information in this process is not merely an objective presentation of food appearance; its specific presentation method influences consumers’ processing of visual food characteristics and the resulting perceptual judgments [7]. In digital food presentations, this visual judgment can further manifest as consumers’ perception of visual realism and visual vividness. Visual realism reflects consumers’ perception of the degree of consistency between the digital presentation and the actual food [8], while visual vividness embodies the clarity and intensity of food color, texture, form, and detail in perception. More importantly, food perception exhibits distinct cross-modal characteristics; visual cues such as color and shape can establish cross-modal links with specific tastes and flavors [5], and visual texture can similarly convey information related to basic tastes [9]. These visual cues can thus form corresponding gustatory expectations prior to actual tasting and influence consumers’ judgments of food sensory attributes [10]. Consequently, visual information in online food not only affects what consumers “see” but also enables them to form internal sensory representations related to food taste in the absence of actual tasting [11]. This cognitive activity extends beyond imagining “how it tastes” and may further involve mental simulation of the consumption process and its outcomes [6]. In this study, these visual-to-sensory processes are considered within a visual compensation framework, which describes how available visual cues support sensory-related judgments when direct access to taste, smell, and touch is constrained in online food consumption.

However, existing research still offers scope for further deepening the explanation of this process. Previous studies have largely focused on the impact of characteristics such as food image type [12], menu visual appeal [13], and dynamic visual presentations [14] on food evaluation and purchase intention. Although these studies demonstrate the importance of visual information, the perceptual, representational, and evaluative processes involved in visual-to-sensory processing have often been examined separately. Consequently, less attention has been paid to how these conceptually distinct processes are related within the sensory-constrained context of online food consumption. Specifically, visual realism and visual vividness capture different properties of visual perception, whereas gustatory mental imagery and texture mental simulation represent different forms of internal sensory representation, and taste expectation reflects an anticipatory evaluation of potential food experience. Therefore, it is necessary to further differentiate these perceptual, representational, and evaluative components and examine their relationships to explain how consumers form judgments about potential food experiences based on visual information.

Furthermore, online food purchase intention may not be formed through a single psychological pathway. Traditional symmetrical analysis primarily identifies the average net effect of variables on outcomes, but consumer decisions are heterogeneous, and the same purchase outcome can arise from different combinations of perceptual and psychological conditions. Existing food consumption research has found that multiple different but equivalent condition configurations can collectively lead to higher purchase intention [15]. Thus, merely answering “which factors significantly influence purchase intention” is insufficient to fully explain the relationships among the perceptual, representational, and evaluative components considered within the visual compensation framework. A configurational perspective can further examine whether different combinations of these conditions are associated with similar levels of purchase intention and whether the configurations associated with high and not high PI differ. Integrating net effects with a configurational perspective can therefore provide a more comprehensive account of heterogeneity in online food purchase judgments.

Based on this, the current study, focusing on the visual compensation framework in online food consumption, constructs a theoretical model comprising Sensory Information Richness (SIR), Visual Realism (VR), Visual Vividness (VV), Gustatory Mental Imagery (GMI), Texture Mental Simulation (TMS), Taste Expectation (TE), and Purchase Intention (PI). PLS-SEM is employed to examine the hypothesized net associations among these constructs, while IPMA evaluates their relative importance and performance with respect to purchase intention. fsQCA further complements the symmetric analysis by identifying alternative configurations of perceptual, representational, and evaluative conditions associated with high and not high PI. Together, these approaches enable the study to examine visual compensation from both structural and configurational perspectives.

## 2. Theoretical Framework and Research Hypotheses

### 2.1. Visual Compensation in Online Food Consumption

Food consumption possesses characteristic multisensory attributes; a consumer’s experience of food is not formed by a single sense but is jointly influenced by multiple sensory inputs, including visual, gustatory, olfactory, and tactile. Furthermore, cross-modal links exist between different senses; visual attributes such as the color and shape of food can impact a consumer’s perception and judgment of taste and flavor [5]. Visual texture can also convey information related to the sensory attributes of food and establishes systematic cross-modal correspondences with basic tastes like sweet, sour, salty, and bitter [9]. Consequently, visual information in food serves not only to identify product appearance but also to provide cues from which consumers may infer sensory attributes beyond vision.

However, online food consumption alters the modalities through which multisensory information is acquired. Sensory interactions in traditional online environments primarily rely on vision and, to a lesser extent, audition, while taste, smell, and touch are difficult to obtain directly prior to purchase [2]. Liem et al. further indicated a notable absence of sensory information in online food selection, as the digital environment in which consumers are situated cannot fully provide the sensory stimulation of an actual eating scenario. In this context, vision becomes a crucial source of information for consumers to infer the sensory attributes of food [3]. Visual features of food images, such as color, can alter a consumer’s judgment of freshness and deliciousness, thereby influencing their consumption response through visually induced gustatory imagery [4]. The visual presentation of digital food may also support consumers’ mental simulation of potential eating processes and outcomes, which may subsequently be associated with purchase intention [6].

Based on the aforementioned research, this study conceptualizes visual compensation as an integrative framework for understanding how consumers use available visual information to form sensory-related judgments when direct gustatory, olfactory, and tactile information is constrained in online food consumption. Compensation does not imply that vision can replicate or entirely substitute for other senses but emphasizes that consumers can leverage visual cues and their cross-modal links to form representations of sensory experiences that are not directly accessible. Cross-modal sensory correspondence provides the perceptual basis for inferring nonvisual sensory attributes from visual cues, whereas sensory imagery and mental simulation describe different forms of internally represented sensory and consumption-related experiences. Visual compensation does not replace these established mechanisms but integrates them within the specific informational condition of online food consumption. Therefore, rather than constituting an additional psychological construct, visual compensation provides a framework linking external visual information with visual perception, internal sensory representation, and anticipatory evaluation. At a broader theoretical level, this framework is consistent with the general logic of the Stimulus–Organism–Response (S-O-R) paradigm, while the four-layer structure adopted here further differentiates visual perception, internal sensory representation, and subsequent evaluation within the specific context of visual compensation in online food consumption.

### 2.2. Sensory Information Richness and Visual Perception

Visual compensation for online food is primarily contingent upon the extent to which digital representations provide information relevant to the food’s sensory attributes. Due to the inability of consumers to directly interact with the product, digital interfaces must employ images, videos, and other visual presentations to convey product information that would typically require direct perception. Existing research on online consumption has characterized these presentations as “sensory-enabling presentations,” which aid consumers in forming perceptions and understanding of products by enhancing the accessibility of product details and sensory attributes [16]. Compared to static presentations with limited information, a higher degree of sensory-enabling presentation can improve consumers’ ability to learn and judge product characteristics [17]. Following this logic, this study defines “Sensory Information Richness” (SIR) as the extent to which online food presentations provide consumers with information pertinent to the food’s sensory attributes. For food items, this information can manifest as attributes such as color, form, surface condition, internal structure, and texture, which can be inferred through digital visual cues.

Sensory information richness (SIR) may first impact consumers’ perception of visual realism (VR). VR emphasizes the degree of visual correspondence between digital representations and consumers’ perceived actual states of food. As online food cannot be directly verified, consumers rely on available visual cues to ascertain whether digital presentations accurately reflect the food’s true state. Gouton et al. found that consumers can judge the visual correspondence between real and virtual food based on specific attributes such as brightness, color, shape, and surface characteristics [8]. This indicates that visual realism is not derived from a single appearance feature but is established through consumers’ comprehensive judgment of multiple visible food attributes. When online presentations provide richer sensory-relevant information, consumers have more basis for judging the actual state of the food, thereby enhancing the perceived consistency between digital and real food representations. Therefore, the following hypothesis is proposed:

**H1.**  *Sensory information richness positively influences visual realism.*

SIR may also influence visual vividness (VV). Unlike VR, which emphasizes the correspondence between visual information and the actual state of food, VV concerns the extent to which food characteristics are perceived in a clear, vivid, and concrete manner. Thus, VR concerns the perceived correspondence of the presented food to its plausible actual state, whereas VV concerns the clarity and vividness of the visual representation that the presentation evokes in the consumer’s mind. A presentation may therefore evoke a vivid visual representation without necessarily being perceived as highly realistic, while a realistic presentation may not necessarily evoke an equally vivid mental representation. Research on food images has shown that visual presentation can alter consumers’ perceptions of product vividness. Meersseman et al. found that different viewing angles in food images produce varying levels of product vividness, which in turn influences food choices [18]. Subsequent research further revealed that the state in which food is depicted in images can also significantly alter product vividness, indicating that the organization of visual food information affects the extent to which consumers form concrete perceptual representations [19]. Accordingly, when online food presentations provide more comprehensive and discernible sensory cues, consumers can obtain more information about the food’s appearance, structure, and texture, enabling them to form more vivid and concrete visual representations of its characteristics. Therefore, the following hypothesis is proposed.

**H2.**  *Sensory information richness positively influences visual vividness.*

### 2.3. Visual Perception and Internal Sensory Representation

Within the visual compensation framework, visual perceptions provide information from which consumers may form internal representations of food experiences that are not directly accessible. Food perception is characterized by cross-modal features; visual cues enable consumers to infer potential tastes and textures before actual tasting [5]. Such internal representations draw on consumers’ prior sensory experiences and the visual information currently available. Consumers can generate mental imagery related to flavor and texture based on visual food cues [11,20]. Therefore, this study further distinguishes between gustatory mental imagery (GMI) and texture mental simulation (TMS). The former reflects consumers’ internal representations of taste attributes in the absence of actual taste stimulation, while the latter emphasizes consumers’ mental simulation of food texture, mouthfeel, and related eating sensations. In this sense, TMS represents a texture-focused form of mental simulation rather than the broader simulation of an entire eating or consumption episode, which may encompass additional aspects of the consumption process and its outcomes.

Visual realism can provide a credible perceptual basis for this internal sensory processing. When digital representations closely resemble the actual food states familiar to consumers, visual cues such as color, shape, surface structure, and internal state are more easily associated with existing food experiences and can be used to infer potential taste and texture experiences. Food images can activate sensory imagery related to taste [4], and neurocognitive studies have found shared neural representations for taste when viewing food images with specific taste attributes and actively imagining the corresponding flavors [21]. Concurrently, consumers can form texture-specific mental imagery based on visual food cues [11]. Higher VR may increase the perceived credibility of visual cues for sensory inference, thereby providing a stronger basis for gustatory mental imagery and textural mental simulation. Therefore, the following hypotheses are proposed:

**H3.**  *Visual realism positively influences gustatory mental imagery.*

**H4.**  *Visual realism positively influences texture mental simulation.*

Visual vividness, in turn, can provide a perceptual basis for internal sensory representation by increasing the clarity and concreteness of food’s visual characteristics. A clear and concrete visual presentation accentuates properties such as color, form, texture, and structure, thereby facilitating the extraction and psychological processing of information related to taste and texture. A systematic cross-modal correspondence exists between visual texture and gustatory perception [9], and visual cues of food can further support consumers in generating flavour and texture mental imagery [20]. When food’s visual characteristics are more vivid and specific, cues associated with potential gustatory and textural experiences may become more accessible for internal sensory processing, thereby providing a stronger basis for GMI and TMS. Therefore, the following hypotheses are proposed:

**H5.**  *Visual vividness positively influences gustatory mental imagery.*

**H6.**  *Visual vividness positively influences texture mental simulation.*

### 2.4. Internal Sensory Representation, Taste Expectation, and Purchase Intention

The internal sensory representations formed through visual compensation further inform consumers’ judgments of future food experiences. Taste expectation (TE) reflects consumers’ anticipatory judgments of the gustatory experience they are likely to have with a food product prior to actual consumption. Unlike GMI, which concerns the mental generation of taste-related sensory representations in the absence of actual tasting, TE concerns an anticipatory evaluation of the likely gustatory experience of the food. Thus, GMI represents what consumers can mentally imagine about taste, whereas TE reflects what they expect the food to taste like when actually consumed. Research by Piqueras-Fiszman & Spence demonstrates that consumers can form sensory expectations based on food cues acquired prior to purchase, and subsequently predict the taste, texture, and other sensory attributes of the food [22]. In this study, GMI provides the internal representation of gustatory attributes for such judgments, while TMS renders potential food experiences more concrete by simulating texture, mouthfeel, and related eating sensations. Furthermore, mental simulation related to food can significantly impact expected taste perception [23]. Although GMI and TMS represent different forms of internal sensory processing, both can provide a psychological basis for consumers to form taste expectations. Therefore, the following hypotheses are proposed:

**H7.**  *Gustatory mental imagery positively influences taste expectation.*

**H8.**  *Texture mental simulation positively influences taste expectation.*

TMS may also directly influence purchase intention. When consumers can simulate the potential texture and mouthfeel of a food product, they may form a more concrete representation of aspects of the potential consumption experience, which can help them assess whether the product aligns with their sensory preferences. Research by Petit et al. indicates that psychological simulation of the potential eating process and outcomes can further enhance purchase intention [6]. Although this general eating simulation is not entirely identical to the texture-focused mental simulation investigated in this study, it underscores that simulated processing related to food experiences can directly inform consumer decision-making. Therefore, the following hypothesis is proposed:

**H9.**  *Texture mental simulation positively influences purchase intention.*

TE, in turn, can further translate consumers’ judgments of potential food experiences into purchase intention. In the absence of actual tasting prior to purchase, consumers need to evaluate the potential consumption value of a food product based on their expected sensory experience. Research on food packaging has found that sensory expectations of a food’s taste and texture can significantly predict purchase intention and play a crucial psychological translation role between visual information and purchase intention [24]. A more positive taste expectation implies that consumers anticipate a more desirable sensory experience from the food, thereby increasing their purchase intention. Therefore, the following hypothesis is proposed:

**H10.**  *Taste expectation positively influences purchase intention.*

### 2.5. Conceptual Model

**Figure 1:**
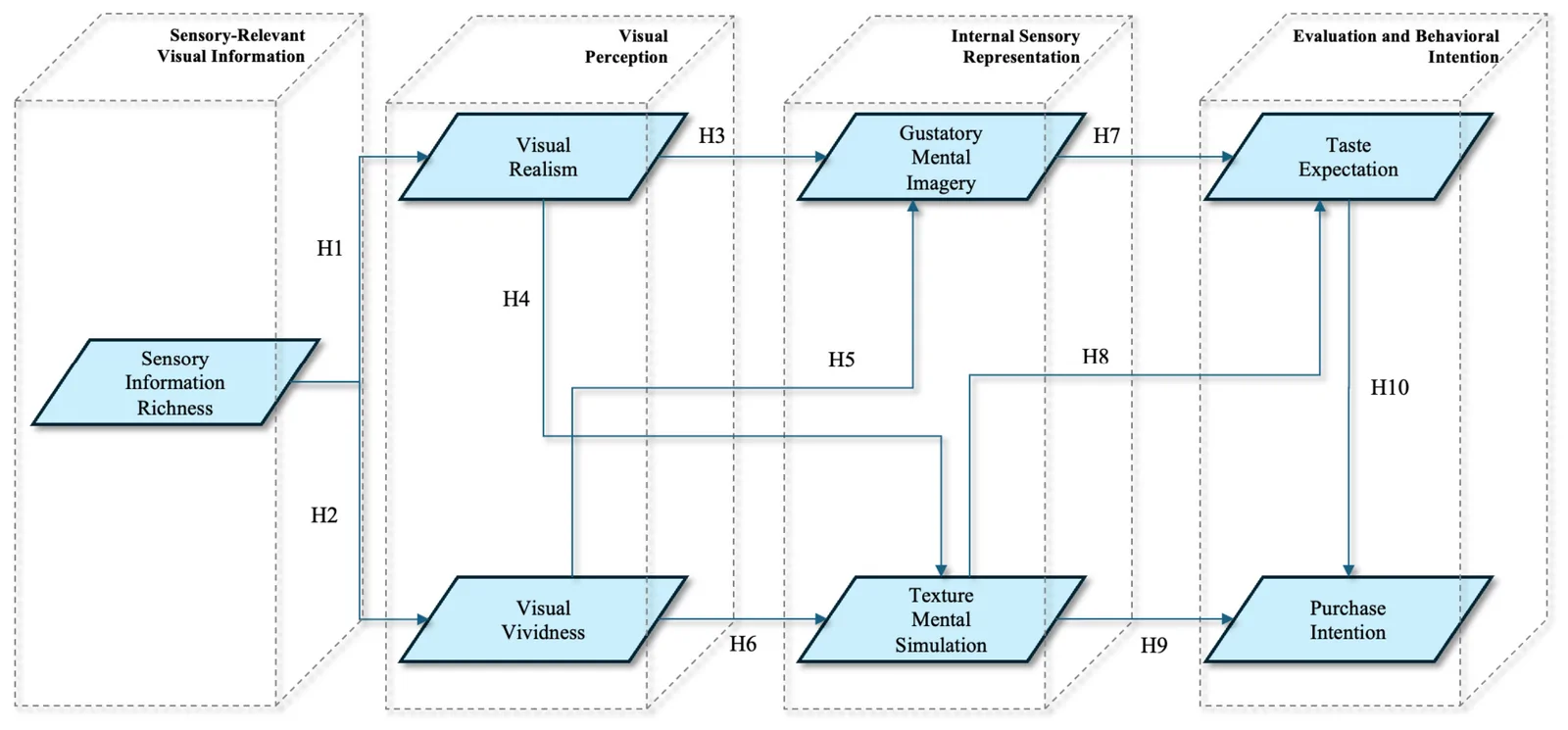
Research model.

Grounded in the theoretical analysis above, this study conceptualizes online food visual compensation as an integrative framework linking external sensory-relevant visual information with visual perception, internal sensory representation, anticipatory evaluation, and purchase intention. Sensory Information Richness represents the sensory-relevant information available in online food presentations, while Visual Realism and Visual Vividness capture distinct aspects of consumers’ visual perception. Gustatory Mental Imagery and Texture Mental Simulation represent different forms of internal sensory representation, while Taste Expectation reflects consumers’ anticipatory evaluation of potential gustatory experience. Purchase Intention represents the subsequent behavioral intention considered in the model. Accordingly, the proposed relationships examine how these perceptual, representational, and evaluative components are associated within the visual compensation framework. This model meticulously distinguishes the differing perceptual functions of Visual Realism and Visual Vividness, as well as the distinct psychological roles of Gustatory Mental Imagery, Texture Mental Simulation, and Taste Expectation within the visual compensation framework. Figure 1 presents the proposed conceptual model.

## 3. Data and Methodology

### 3.1. Variable Measurement

This study employed multi-item scales to measure the seven constructs within the research model. The measurement items were developed or contextually adapted with reference to existing research and aligned with the construct definitions and online food consumption context of this study. The measurement of Sensory Information Richness (SIR) was informed by Jai et al. [16], and Visual Realism (VR) by Gouton et al. [8]. Visual Vividness (VV) and Gustatory Mental Imagery (GMI) were contextually adapted from relevant dimensions of the Plymouth Sensory Imagery Questionnaire developed by Andrade et al. [25]. The measurement of Texture Mental Simulation (TMS) references Petit et al. [6], Taste Expectation (TE) references Garaus et al. [26], and Purchase Intention (PI) references Liu et al. [12]. Where direct item transfer was not appropriate, the referenced literature was used to inform item development and contextual adaptation rather than being treated as providing an original validated scale for the present construct. Each item was rephrased to align with the context of viewing food images and online consumption scenarios, thereby ensuring consistency between the measurement content and construct definitions. No standardized food image or researcher-controlled visual stimulus was presented during the survey. Respondents evaluated the constructs with reference to their general experiences of viewing food images when browsing or purchasing food online. Accordingly, the measures capture consumers’ generalized perceptions and self-reported mental responses associated with online food visual presentations rather than responses to a specific standardized stimulus. All items were assessed using a 7-point Likert scale, where 1 indicated “strongly disagree” and 7 indicated “strongly agree.” The construct definitions, English measurement items, and corresponding sources are reported in Table 1, while the Chinese version of the measurement items is provided in Appendix A.

### 3.2. Sample and Data Collection

The formal questionnaire was constructed based on the aforementioned measurement items and comprised three primary sections: study instructions, respondent demographic information, and core construct measurement items. The study instructions outlined the research objectives, completion requirements, and principles of anonymity and voluntary participation. The demographic section included questions on gender, age, education level, and monthly online food purchase frequency. The core measurement section was designed to assess the seven constructs investigated in this study: Sensory Information Richness (SIR), Visual Realism (VR), Visual Vividness (VV), Gustatory Mental Imagery (GMI), Texture Mental Simulation (TMS), Taste Expectation (TE), and Purchase Intention (PI).

This study targeted adult consumers aged 18 years or older with prior experience in browsing or purchasing food online. Data collection was conducted via online questionnaires from April to July 2026. A non-probability sampling approach was adopted, and the questionnaire was administered through the Wenjuanxing online survey platform. Participants were recruited through both the platform’s respondent distribution channels and questionnaire links distributed by the research team. No monetary or material incentives were provided for participation. Respondents evaluated online food visual presentations and their associated perceptual, sensory-imagery, and purchase responses based on their prior online food browsing and purchasing experiences. Prior to completing the questionnaire, all participants were informed about the research objectives, anonymity, voluntary participation, and their right to withdraw from the study at any time. A screening question was included to confirm whether participants had experience browsing or purchasing food online; those who did not meet the screening criteria were excluded from further participation.

Following data collection, a quality check of the returned questionnaires was performed, focusing on completeness, completion time, and response patterns. Questionnaires exhibiting signs of rapid or patterned responding, along with other indicators of low quality, were excluded. A total of 1375 questionnaires were initially collected. Responses with incomplete information, unusually short completion times, or evident repetitive or invariant response patterns were excluded during data screening. After excluding 149 responses that did not meet the eligibility or quality-control criteria, a total of 1226 valid questionnaires were obtained, and the data used for subsequent analysis were free of missing values. Table 2 presents the demographic characteristics of the respondents. Among the 1226 respondents, 638 were male (52.04%) and 588 were female (47.96%), indicating a relatively balanced gender distribution. The age distribution spanned various age groups, with the 21–30 age bracket representing the largest proportion (24.06%), and all other age groups comprising at least 17% of the sample. In terms of educational attainment, respondents with a bachelor’s degree or lower, master’s degree, and doctoral degree or higher accounted for 46.82%, 35.81%, and 17.37%, respectively. Regarding monthly online food purchase frequency, 24.06% of respondents purchased 1–2 times, 32.06% purchased 3–5 times, 29.04% purchased 6–10 times, and 14.85% purchased more than 10 times. These characteristics indicate variation in respondents’ demographic backgrounds and online food purchasing frequency.

### 3.3. Research Methods

This study employed PLS-SEM, IPMA, and fsQCA for data analysis. PLS-SEM and IPMA were performed using SmartPLS 4.1.1.8. PLS-SEM was first used to assess the measurement and structural models and to examine the hypothesized net associations among the constructs in the proposed model. The significance of the structural relationships was evaluated through bootstrapping. Subsequently, IPMA was applied to assess the relative importance and performance levels of each antecedent construct with respect to purchase intention, thereby identifying constructs that combine relatively high importance with potential for performance improvement. Building upon this, fsQCA was used to analyze the conditional configurations of high PI versus not high PI, thereby identifying alternative combinations of conditions associated with the same outcome and examining whether the configurations associated with high and not high PI differ. The integration of these three methods provides a combined assessment of structural relationships, relative importance and performance, and configurational patterns in the proposed model.

## 4. Results

### 4.1. Common Method Variance Test

To ensure the accuracy of statistical analysis results, this study employed the widely adopted Harman’s single-factor test to examine common method variance in the data [27]. The test results, presented in Table 3, indicated the extraction of seven common factors with eigenvalues greater than 1, accounting for a cumulative variance of 74.601%. The first factor explained 31.584% of the variance, which is below the 40% threshold, suggesting that no single factor dominates the variance. As an additional assessment, full collinearity VIFs were calculated for all seven constructs. The values ranged from 1.150 to 1.549, all below the recommended threshold of 3.3 [28], providing further evidence that common method bias is unlikely to be a major concern in the present data.

### 4.2. Assessment of Measurement Model

Table 4 presents the reliability and validity test results. The external loadings of all measurement items exceeded the reference value of 0.7. The Cronbach’s α coefficients for all constructs were above 0.7 [29], composite reliability (CR) values were above 0.7 [30], and average variance extracted (AVE) values were above 0.5 [31]. These findings indicate satisfactory indicator reliability, internal consistency reliability, and convergent validity of the measurement model.

Two standard criteria were adopted to assess discriminant validity. The HTMT ratios (Table 5) were all below 0.85 [32]. According to the Fornell–Larcker criterion (Table 6), the square roots of the AVE (bolded diagonal elements) were greater than the inter-construct correlations in their respective rows and columns [33]. Therefore, both criteria support adequate discriminant validity among the seven constructs.

### 4.3. Assessment of Structural Model

Table 7 reports the explanatory power and predictive relevance of the structural model. The R^2^ values for the endogenous variables ranged from 0.020 to 0.214. Specifically, the R^2^ values for GMI, PI, TE, and TMS were 0.151, 0.182, 0.214, and 0.158, respectively, while the R^2^ values for VR and VV were 0.025 and 0.020. Furthermore, the Q^2^ predictive values for all endogenous variables were greater than 0, ranging from 0.017 to 0.057, indicating out-of-sample predictive relevance across the endogenous constructs.

The research hypotheses were tested using the bootstrapping method. Bootstrapping was performed using 5000 subsamples, two-tailed testing, and 95% percentile confidence intervals. Table 8 presents the standardized path coefficients, *p*-values, VIFs, and hypothesis testing results for each path. The results indicate that all hypothesized paths achieved statistical significance and were supported. Specifically, SIR had a significant positive effect on both VR (β = 0.158, *p* < 0.001) and VV (β = 0.143, *p* < 0.001), supporting H1 and H2. VR significantly positively influenced GMI (β = 0.164, *p* < 0.001) and TMS (β = 0.161, *p* < 0.001), supporting H3 and H4; VV also exhibited significant positive effects on GMI (β = 0.312, *p* < 0.001) and TMS (β = 0.325, *p* < 0.001), supporting H5 and H6. Further analysis revealed that GMI had a significant positive effect on TE (β = 0.369, *p* < 0.001), supporting H7. TMS exerted significant positive effects on PI (β = 0.208, *p* < 0.001) and TE (β = 0.188, *p* < 0.001), supporting H9 and H8, respectively. The positive effect of TE on PI also reached a significant level (β = 0.315, *p* < 0.001), supporting H10. Additionally, the VIF values for all paths ranged from 1.000 to 1.106, all below the common judgment standard [34], indicating no significant multicollinearity issues in the structural model. The f^2^ values ranged from 0.021 to 0.157, with the GMI–TE relationship reaching a medium effect size (f^2^ = 0.157).

The specific indirect effects were further examined using the same bootstrapping procedure. GMI showed a significant indirect association with PI through TE (β = 0.116, 95% CI [0.082, 0.154]), while TMS was also indirectly associated with PI through TE (β = 0.058, 95% CI [0.037, 0.080]). At the visual level, significant indirect associations with PI through GMI and TE were observed for both VR (β = 0.019, 95% CI [0.011, 0.028]) and VV (β = 0.036, 95% CI [0.023, 0.053]). VR and VV were also indirectly associated with PI through TMS (VR: β = 0.033, 95% CI [0.017, 0.053]; VV: β = 0.067, 95% CI [0.040, 0.098]). These results further support the indirect pathways linking visual perceptions, internal sensory representations, taste expectation, and purchase intention.

### 4.4. Importance–Performance Map Analysis

IPMA is a method based on PLS-SEM analysis that simultaneously examines the importance and performance levels of various constructs. The horizontal axis represents the total effect of each antecedent construct on the target construct, indicating construct importance, while the vertical axis represents the performance level of each construct, standardized to a 0–100 scale [35]. By comprehensively comparing construct importance and performance levels, IPMA can further differentiate constructs according to their relative importance and current performance, thereby providing a basis for identifying potential priorities within the proposed model.

**Figure 2:**
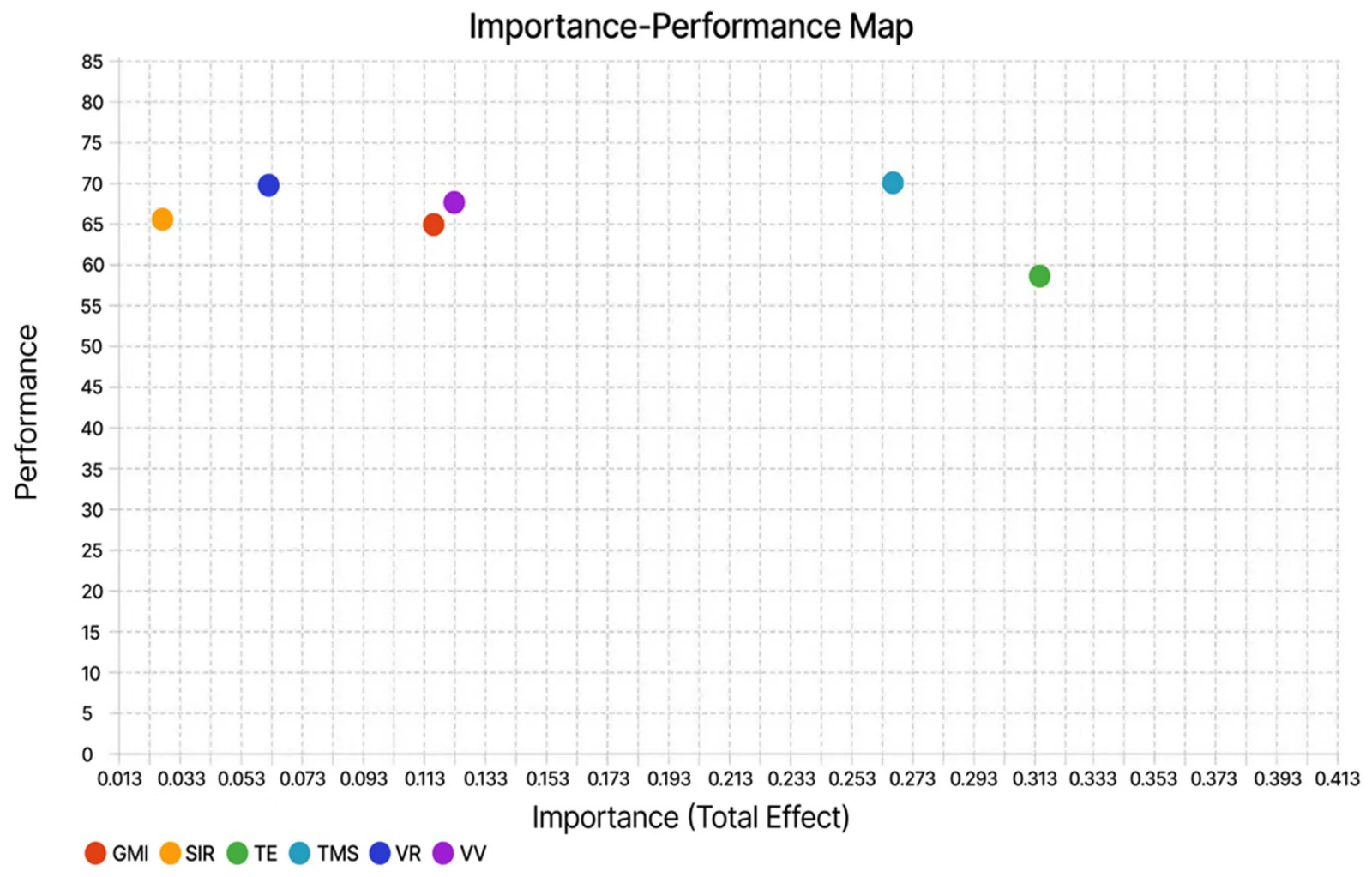
Importance–performance map for purchase intention (PI).

Figure 2 presents the IPMA results for each construct. In terms of importance, TE exhibits the highest importance, followed by TMS, indicating that these two constructs have the largest total effects on PI among the constructs included in the analysis. Specifically, TE’s importance is approximately 0.31, but its performance level is around 59, which is relatively low among all constructs, displaying a distinct “high importance–relatively low performance” characteristic. This positions TE as a relatively high-priority construct in the importance–performance comparison. TMS has an importance of approximately 0.27 and a performance level of around 70, exhibiting a “high importance-high performance” characteristic, reflecting its relatively high total effect on PI together with a comparatively high performance level.

In contrast, GMI and VV are at a moderate level of importance, with performance levels exceeding 65. VV’s performance is slightly higher than GMI’s, suggesting that although they are not the most important constructs for PI in the IPMA results, they still show moderate relative importance. VR and SIR have relatively low importance. Despite their high performance levels, their total effect on PI is limited. Therefore, considering both importance and performance dimensions, TE represents a potential priority, while TMS combines relatively high importance with high performance. GMI and VV occupy intermediate positions in the importance–performance comparison, whereas VR and SIR show relatively lower importance.

### 4.5. Fuzzy-Set Qualitative Comparative Analysis

The analysis of the formation mechanism of online food purchase intention requires not only examining the net effect relationships between variables but also further considering the potential joint and substitutive patterns among multiple conditions. Traditional PLS-SEM is primarily used to test the linear net effects between variables. In contrast, fsQCA can identify configurations of conditions associated with an outcome from a configurational perspective. Therefore, this study further employs fsQCA to conduct configurational analysis for the formation mechanisms of high purchase intention (High PI) and not high purchase intention (Not High PI) to identify alternative combinations of conditions associated with these outcomes.

Prior to the configurational analysis, this study first calibrated each condition variable and outcome variable and conducted a necessity analysis. Using the direct calibration method, the 95th, 50th, and 5th percentiles of the data distribution for each variable were used as anchors for full membership, crossover point, and full non-membership, respectively, transforming the raw data into fuzzy-set membership degrees between 0 and 1. Subsequently, using high PI and not high PI as outcomes, necessity tests were performed for SIR, VR, VV, GMI, TMS, and TE and their negated states. A consistency value of 0.90 or higher was adopted as the criterion for identifying a necessary condition [36].

As shown in Table 9, for high PI, the consistency of each single condition and its negated state ranges between 0.589 and 0.765. GMI has the highest consistency (0.765) but still does not meet the necessary condition judgment standard of 0.90. For not high PI, the consistency of each condition and its negated state ranges between 0.517 and 0.785. Among these, ~GMI has the highest consistency (0.785), which is also below 0.90. This indicates that neither for high purchase intention nor for not high PI do any antecedent variables individually constitute a necessary condition for the outcome. Accordingly, the analysis proceeded to examine sufficient configurations involving combinations of conditions associated with high and not high PI.

Building upon the necessity analysis, this study further employed truth table analysis to identify the condition combinations associated with high PI versus not high PI. Following the recommendations of Kraus et al. [37], the raw consistency threshold was set at 0.80, the PRI consistency threshold at 0.70, and the case frequency threshold at 3. In determining the configuration results, core and peripheral conditions were identified by comparing the parsimonious and intermediate solutions. Conditions appearing in both the parsimonious and intermediate solutions were considered core conditions, while those appearing only in the intermediate solution were deemed peripheral conditions.

As presented in Table 10, nine configurations (M1–M9) were identified for high PI, with individual configuration consistency ranging from 0.867 to 0.914, all exceeding the judgment criterion of 0.80. M9 exhibited the highest consistency at 0.914. The raw coverage for each path ranged from 0.390 to 0.526, with an overall solution coverage of 0.732 and an overall solution consistency of 0.818, indicating that the identified configurations provide substantial coverage of high PI with satisfactory overall consistency. A robustness check using a higher frequency threshold of 5 yielded six configurations, with an overall solution coverage of 0.693 and consistency of 0.834.

Based on the core conditions and their combined characteristics, the nine high PI configurations could be further categorized into four patterns. The first category is the VR-GMI configuration (M1, M3, M4), primarily characterized by the presence of VR and GMI. The second category is the VV-GMI configuration (M2, M5, M7), characterized by the presence of VV and GMI under different combinations of other conditions. The third category is the GMI-TE configuration (M6, M8), in which GMI and TE occur together with different accompanying conditions. The fourth category is the multi-condition configuration (M9), which combines several perceptual and psychological conditions within a single configuration. Overall, GMI appears in most configurations associated with high PI configuration; however, the necessary condition analysis revealed that it is not a necessary condition for high PI. The presence of multiple configurations associated with the same outcome indicates configurational equifinality in high PI.

As illustrated in Table 11, eight configurations (N1-N8) were identified for not high PI, with configuration consistency ranging from 0.750 to 0.865 and raw coverage ranging from 0.580 to 0.785. The overall solution coverage was 0.945, and the overall solution consistency was 0.692. Although these configurations demonstrated high overall coverage, their overall consistency was relatively low and lower than that of the high PI solution. Therefore, the not high PI results are interpreted cautiously and are used primarily as a supplementary comparison with the configurations associated with high PI.

In terms of condition combinations, not high PI is more characterized by various forms of missing combinations of SIR, VR, VV, GMI, TMS, and TE. However, the absence of any single condition could not explain all not high PI configurations. The not high PI configurations therefore suggest heterogeneous combinations of absent and present conditions rather than the absence of a single condition. More importantly, the configurations of high and not high PI did not exhibit a simple mirror image relationship. Given the relatively low overall consistency of the not high PI solution, however, this contrast should be interpreted cautiously rather than as conclusive evidence of causal asymmetry. Consequently, beyond the net effects between variables revealed by PLS-SEM, fsQCA identifies multiple configurations associated with high PI and provides supplementary evidence on the distinct configurational patterns associated with not high PI.

## 5. Discussion

### 5.1. Visual Decoding of Sensory Information in Online Food Consumption

This study first elucidates the upstream process of the conversion of sensory information to visual perception in online food consumption. SIR positively and significantly impacts both VR and VV, supporting H1 and H2. This indicates that when direct sensory experiences of food are not readily accessible, consumers can process available sensory information through the visual channel rather than simply receiving the food’s visual presentation. This aligns with Yeh et al.’s findings, which suggest that consumers’ understanding of food images involves multiple perceptual dimensions such as informativeness, aesthetics, and color [38]. Furthermore, the richness of sensory information enhances consumers’ perception of food visual authenticity and visual vividness, making visual presentation a crucial entry point for understanding the sensory attributes of online food.

The simultaneous confirmation of H1 and H2 further reveals the differential mechanisms of this visual decoding. VR focuses on the consistency between the digital representation and the actual food state, echoing previous research on the perceptible correspondence between visual representations of real and virtual food [8]. In contrast, VV emphasizes whether food features can form clear, distinct, and concrete perceptual representations, suggesting that rich sensory information can also enhance the perceptual intensity of food’s visual characteristics. Therefore, VR and VV are not redundant reflections of the same visual effect, but rather correspond to the perceived correspondence between the visual presentation and the food’s actual state and the vividness of the resulting visual representation, respectively. Thus, visual compensation does not involve direct visual substitution for gustatory perception, but rather the use of visual information to support different forms of perceptual representation, which provide a basis for subsequent cross-modal psychological processing.

### 5.2. Psychological Transformation from Visual Perception to Purchase Intention

Upon completing the visual decoding of sensory information, both VR and VV further positively influence GMI and TMS, supporting H3–H6. This indicates that the visual perception of online food can advance from external information recognition to internal sensory representation. Existing research suggests that consumers can form mental imagery related to flavor and texture based on food images [11], and visual food cues can be directly used to generate flavor and texture mental imagery [20]. This internal representation is driven by both VR and VV, with the estimated path coefficients from VV to GMI and TMS being numerically larger than those from VR. This implies that in an online environment lacking direct sensory feedback, visual compensation may not only depend on the similarity between food presentation and the actual object but also on whether visual cues can form vivid, concrete, and easily evoked perceptual experiences. In other words, “looking authentic” provides a credible basis for sensory inference, while “looking vivid” may be more conducive to translating these cues into mental experiences of taste and texture.

GMI and TMS also exhibit distinct psychological translation functions. GMI significantly promotes TE, while TMS not only promotes TE but also directly influences PI, supporting H7, H8, and H9. Visual food cues can evoke gustatory mental imagery and further participate in consumer evaluation and behavioral judgment, which aligns with the findings of Thomas & Capelli’s research where visual flavor cues influenced purchase intention through flavor mental imagery [39]. Concurrently, visual food cues can also alter consumers’ expected taste perception through mental simulation [23]. This study further differentiates these two types of internal processing: GMI primarily forms internal representations of the potential gustatory attributes of food, whereas TMS focuses on the simulation of texture, mouthfeel, and related eating sensations. Notably, TMS’s direct effect on PI suggests that when consumers can simulate more concrete food experiences mentally, texture-focused simulation may contribute to purchase judgment beyond its relationship with taste expectation. This finding is consistent with broader eating-simulation research showing that mentally simulating the eating process or its outcomes can inform consumer decision-making [6]. Therefore, GMI and TMS are not redundant mediating processes but rather undertake gustatory representation and experiential simulation functions, respectively, participating in subsequent decision-making in different ways.

The significant positive effect of TE on PI further completes the psychological translation from visual perception to purchase judgment, supporting H10. Consumers do not directly form purchase judgments based solely on visual food stimuli but rather base them on the gustatory imagery and texture simulation activated by visual cues, forming expectations about the unexperienced eating experience. The external visual cues of food can systematically shape consumers’ sensory expectations, and these expectations serve as an important basis for evaluating food prior to the actual experience. Thus, the visual compensation elucidated in this study is not a direct substitution of vision for taste or touch, but rather a cross-modal psychological translation process that activates internal sensory representations through visual perception, forms expected experiences, and subsequently leads to purchase judgments.

### 5.3. Priority Mechanisms Revealed by IPMA

IPMA analysis unveiled disparities in the importance and performance of psychological mechanisms in the posterior stages of visual compensation. Specifically, TE exhibited high importance and relatively low performance, indicating that although taste anticipation is a critical psychological juncture driving purchase intention, TE represents a potentially important area for improvement within the visual compensation process. Improving the extent to which visual information supports clear and realistic anticipated taste experiences may therefore hold greater priority value than simply augmenting visual stimuli further. This also suggests that the ultimate impact of visual compensation on purchase judgment depends not only on consumers’ ability to form internal representations related to taste but also on whether these representations can be further translated into explicit taste expectations.

In contrast, TMS demonstrated high importance and high performance, suggesting that consumers effectively utilize visual information to simulate food texture and mouthfeel, a well-established psychological processing component. This is corroborated by the findings that TMS can both promote TE and directly influence PI. Therefore, TE and TMS represent distinct priority states within the posterior mechanisms of visual compensation. TE combines relatively high importance with greater potential for performance improvement, while TMS combines high importance with an already relatively high performance level. IPMA thus further differentiates the functional value and improvement priorities of different psychological mechanisms, offering supplementary explanations for how visual compensation can more effectively foster online food purchase intention.

### 5.4. Configurational Mechanisms of Purchase Intention

fsQCA results further indicate that online food purchase intention is markedly configurationally dependent. While PLS-SEM reveals the overall net effect relationship between visual perception, internal sensory processing, and purchase judgment, the configurational analysis demonstrates that high PI does not rely on a singular, fixed psychological pathway. Various combinations of different visual perception and psychological processing conditions can jointly achieve the same outcome. Similar multi-path characteristics have been observed in existing consumer research, where different antecedent conditions can lead to purchase intention through multiple equivalent configurations [40]. This implies that although visual compensation follows an overall pattern of transformation from visual perception to internal sensory experience and purchase judgment, consumers do not necessarily complete this process in an identical manner. Instead, they can leverage different perceptual and psychological resources to form high PI.

This multi-path characteristic further reflects differences in how conditions are combined across configurations. Within different configurations, VR and VV can serve as distinct visual perception foundations, while GMI, TMS, and TE can play differentiated roles in various conditional combinations. Different combinations of visual and psychological conditions may therefore be associated with the formation of high PI. Consequently, the variable relationships uncovered by SEM can coexist with the multiple conditional configurations identified by fsQCA, reflecting the complex interplay among different conditions in the formation of purchase intention. Visual compensation is not a rigid psychological chain applicable to all consumers but rather a configurational process characterized by convergent pathways.

The configurational results also show that the configurations associated with high and not high PI do not exhibit a simple mirror-image pattern. However, given the relatively low overall consistency of the not high PI solution, this difference should be interpreted cautiously rather than as conclusive evidence of causal asymmetry. This aligns with findings in food consumption research where high and low purchase intentions are formed by different antecedent configurations [41]. The presence or absence of a particular visual or psychological condition may take on different roles across configurations, depending on the other conditions with which it occurs. Therefore, the contribution of fsQCA to PLS-SEM lies not only in identifying additional pathways but also in elucidating the configurational complexity underlying net effect relationships. Taken together, these analyses suggest that online food visual compensation exhibits both relatively stable overall psychological transformation patterns and allows consumers to achieve similar purchase judgments through diverse conditional combinations.

## 6. Conclusions and Implications

This study investigates how visual information in online food consumption can compensate for the lack of direct sensory experience. A visual compensation mechanism model was constructed, comprising Sensory Information Richness (SIR), Visual Realism (VR), Visual Vividness (VV), Gustatory Mental Imagery (GMI), Texture Mental Simulation (TMS), Taste Expectation (TE), and Purchase Intention (PI). The model was analyzed using PLS-SEM, IPMA, fsQCA. The findings indicate that visual compensation in online food consumption is not a simple, direct effect of visual stimuli on purchase intention. Instead, the proposed visual compensation framework links sensory-relevant visual information with visual perception, internal sensory representations, taste expectations, and purchase judgments through a series of theoretically specified relationships.

Specifically, SIR simultaneously enhances both VR and VV, suggesting that consumers can initially decode the sensory attributes of online food products through the visual channel. Building upon this, VR and VV further promote GMI and TMS, with the estimated effects of VV on GMI and TMS being numerically larger than those of VR. GMI primarily influences subsequent judgments through TE, while TMS not only reinforces TE but also directly contributes to PI. TE ultimately serves as a crucial downstream component in the conversion of internal sensory processing to purchase judgments.

Further analyses using IPMA and fsQCA expand upon this linear mechanism. TE demonstrates high importance but relatively low performance, indicating greater potential for performance improvement relative to the other constructs. In contrast, TMS exhibits both high importance and high performance, positioning it as a relatively stable advantageous component in the visual compensation process. Concurrently, high PI can be achieved through various combinations of visual perception and psychological processing conditions, demonstrating multiple configurations associated with the same outcome. Furthermore, configurations associated with high PI differ from those associated with Not high PI. Thus, visual compensation in online food consumption exhibits both relatively stable psychological transformation regularities and pronounced configurational complexity.

### 6.1. Theoretical Implications

This research offers theoretical contributions to the study of online food consumption.

1. This study extends research on visual information in online food consumption from the perspective of visual compensation. Moving beyond viewing food’s visual presentation primarily as an external stimulus influencing product evaluation or purchase intention, this research positions it within the context of online sensory experience deficiency. It elucidates how visual information can fulfill a portion of sensory information transmission functions, subsequently activating internal psychological processing related to taste and texture. This perspective broadens the scope of food visual research from mere presentation effectiveness to intersensory psychological transformation processes.
2. This study meticulously details the internal mechanisms by which visual perception contributes to internally represented food sensory experiences. By differentiating between VR and VV, as well as GMI, TMS, and TE, this research demonstrates that external visual information incorporates multiple distinct psychological processing stages before influencing purchase judgments. Notably, the divergent pathways observed for GMI and TMS suggest that consumers’ psychological reconstruction of absent sensory experiences is not a monolithic imaginative process but encompasses different forms, such as sensory representation and experiential simulation.
3. By integrating net effect analysis with priority mechanism and configurational analysis, this study further refines the explanation of the formation mechanism of purchase intention in online food consumption. PLS-SEM reveals stable overall relational effects between variables. IPMA identifies the importance and improvement potential of different psychological stages. fsQCA, in turn, uncovers the possibility of multiple condition combinations leading to identical purchase outcomes. Collectively, these three analytical approaches demonstrate that visual compensation not only involves general psychological transformation regularities but also exhibits configurational characteristics such as configurational complexity and equifinality.

### 6.2. Practical Implications

This research offers practical insights for online food visual design, guided by perceptual compensation.

1. Digital food presentation should shift from solely pursuing visual appeal to enhancing the perceptibility of sensory information. Rich sensory information can enter subsequent psychological processing through visual authenticity and visual vividness, while the visual cues provided should remain consistent with the actual sensory attributes of the food. Therefore, the design focus should not be on merely increasing the number of images or strengthening visual effects, but on selecting visual cues that effectively represent the sensory attributes of food. For attributes such as color, surface texture, internal structure, and mouthfeel, which are difficult to experience directly online, their recognizability can be enhanced through methods like close-ups, cross-sectional views, scale references, and dynamic representations. On the basis of realistic restoration, the visual prominence of key sensory features should be improved.
2. The Taste Expectation (TE) should be prioritized as an optimization element in visual compensation design. The high importance and relatively low performance of TE indicate that although consumers can form taste-related judgments from visual information, there remains potential to improve how effectively visual information supports the formation of taste expectations. Consequently, visual design needs to strengthen the correspondence between external cues and expected taste experiences, enabling consumers not only to see food characteristics but also to form relatively clear taste judgments based on them. Color, texture, processing, and consumption states in images, videos, and interactive displays should maintain informational consistency, reduce perceptual biases caused by excessive embellishment, and thereby improve the effectiveness of the conversion of visual information into taste expectations.
3. The high importance and high performance of Texture Mental Simulation (TMS) suggest that psychological simulation of texture and mouthfeel can become a significant advantage in online food visual design. Based on this finding, digital presentation can further highlight visual processes that represent textural changes, such as cutting, stretching, breaking, flowing, squeezing, and chewing, which may help consumers to anticipate sensory characteristics like crispness, softness, viscosity, juiciness, or fineness. Such designs do not simply add dynamic effects but utilize visually directional information to help consumers form more specific potential eating experiences.
4. The fsQCA results indicate that high PI can be achieved through various condition combinations, suggesting that a completely uniform visual compensation strategy is not advisable for online food design. In practice, because different foods may vary in their salient sensory attributes, visual design can emphasize different types of information according to product characteristics. For foods where external authenticity is more important, realistic restoration and detailed presentation can be strengthened. For foods where textural experience is more prominent, dynamic visual cues and textural information expression should be enhanced. Thus, online food visual design can shift from uniformly strengthening visual stimuli to emphasizing sensory-relevant information according to product characteristics, enabling visual presentation to more effectively serve the perceptual compensation function in online consumption environments.

### 6.3. Limitations and Future Research

This study has certain limitations.

1. This study employed cross-sectional data to examine theoretically grounded relationships between variables. Consequently, the findings primarily reflect the correlational structure among various psychological variables and do not fully elucidate the temporal dynamics of visual compensation processes. Future research could utilize longitudinal surveys or multi-stage experiments to further investigate the dynamic relationships among visual perception, internal sensory representations, and purchasing judgments.
2. This study primarily discussed sensory compensation in online food consumption from the perspective of visual information. However, digital food experiences can also be influenced by auditory cues, linguistic descriptions, interactive feedback, and emerging olfactory and haptic technologies. Future research could further explore the synergy or conflict between visual and other digital sensory cues and compare the roles of different sensory combinations in the reconstruction of food experiences.
3. This study focused on the general psychological mechanisms of visual compensation, whereas different food categories and consumption contexts may entail distinct sensory information requirements. For instance, foods heavily reliant on texture judgments and those primarily assessed by taste or aroma may exhibit divergent visual compensation pathways. Future research could conduct comparative studies incorporating food types, consumer sensory preferences, and digital presentation formats to examine the boundary conditions and applicability of visual compensation mechanisms.

## Figures and Tables

**Table 1:** Construct definitions, measurement items, and sources.

Construct	Definition	Code	Item	Source
Sensory Information Richness (SIR)	The extent to which online food presentations provide sufficient visual information for consumers to infer sensory attributes of the food.	SIR1	The online food images I usually encounter provide rich information about the sensory characteristics of the food.	[16]
		SIR2	These images provide sufficient information for me to judge characteristics of the food, such as its color, condition, and texture.	
		SIR3	These images usually contain multiple visual cues related to the food’s taste, temperature, or mouthfeel.	
Visual Realism (VR)	The extent to which the visual presentation of food is perceived to correspond to its actual appearance and physical state.	VR1	The online food images I usually encounter realistically represent the appearance of the food.	[8]
		VR2	The texture and surface characteristics of the food shown in these images usually appear realistic.	
		VR3	The food shown in these images usually has a visual appearance that closely resembles the actual food.	
		VR4	Details shown in these images, such as gloss, moisture, and surface condition, usually provide a realistic representation of the food’s actual appearance.	
Visual Vividness (VV)	The extent to which a food presentation conveys its visual characteristics in a clear, vivid, and concrete manner.	VV1	When viewing online food images, I can usually form a clear and vivid visual representation of the food in my mind.	[25]
		VV2	Online food images usually allow me to imagine the appearance of the food in a concrete way.	
		VV3	When viewing online food images, I can usually form a vivid visual image of the food in my mind.	
		VV4	I can usually reproduce the visual details of the food shown in the images clearly in my mind.	
		VV5	Online food images usually leave me with a vivid and concrete visual impression of the food.	
Gustatory Mental Imagery (GMI)	The extent to which consumers mentally generate taste-related sensory representations of food without actual tasting.	GMI1	When viewing online food images, I can sometimes almost experience what the food might taste like.	[25]
		GMI2	I can usually imagine taste characteristics such as the sweetness or saltiness of the food based on online food images.	
		GMI3	When viewing online food images, I can form a relatively clear mental representation of the food’s taste.	
		GMI4	Visual information such as sauces and ingredients in the images enables me to imagine what the food might taste like.	
		GMI5	Simply by viewing online food images, I can usually form relatively vivid gustatory imagery of the food.	
Texture Mental Simulation (TMS)	The extent to which consumers mentally simulate the texture, mouthfeel, and related oral sensations of food based on visual information.	TMS1	When viewing online food images, I can imagine textural sensations such as crispness in the mouth.	[6]
		TMS2	Based on online food images, I can imagine textural sensations such as firmness, softness, creaminess, or melting in the mouth.	
		TMS3	I can use visual information to mentally simulate textural experiences such as smoothness or creaminess in the mouth.	
Taste Expectation (TE)	Consumers’ anticipatory judgment of the taste experience that a food is likely to provide before actual consumption.	TE1	Based on online food images, I can usually form expectations about how tasty the food is likely to be.	[26]
		TE2	After viewing online food images, I can usually form a judgment about what the food is likely to taste like.	
		TE3	Based on online food images, I can usually anticipate the taste and mouthfeel experience that the food is likely to provide.	
Purchase Intention (PI)	The extent to which consumers are willing or likely to purchase the food presented online.	PI1	I would be likely to purchase food presented in this way online.	[12]
		PI2	I would be interested in purchasing food presented in this way online.	
		PI3	I would be willing to try food presented in this way online.	
		PI4	If given the opportunity, I would consider adding food presented in this way to my shopping cart.	
		PI5	I would consider purchasing food presented in this way in the near future.	

**Table 2:** Descriptive analysis of respondents (*n* = 1226).

Characteristics	Category	Frequency	Percentage (%)
Gender	Male	638	52.04
	Female	588	47.96
Age	20 and below	233	19.00
	21–30	295	24.06
	31–40	221	18.03
	41–50	258	21.04
	51 and above	219	17.86
Education	Bachelor’s degree or below	574	46.82
	Master’s degree	439	35.81
	Doctoral degree or above	213	17.37
Monthly frequency of online food purchases	1–2 times	295	24.06
	3–5 times	393	32.06
	6–10 times	356	29.04
	More than 10 times	182	14.85

**Table 3:** Common method bias test.

Components	Initial Eigenvalue			Extraction Load Squared Sum		
	Total	Percentage of Variance	Cumulative%	Total	Percentage of Variance	Cumulative%
1	8.844	31.584	31.584	8.844	31.584	31.584
2	2.925	10.448	42.032	2.925	10.448	42.032
3	2.531	9.040	51.072	2.531	9.040	51.072
4	2.021	7.219	58.290	2.021	7.219	58.290
5	1.706	6.091	64.381	1.706	6.091	64.381
6	1.504	5.370	69.751	1.504	5.370	69.751
7	1.358	4.850	74.601	1.358	4.850	74.601

**Table 4:** Measurement model assessment.

Constructs	Items	Loadings	α	CR	AVE
GMI	GMI1	0.877	0.918	0.938	0.752
	GMI2	0.868			
	GMI3	0.860			
	GMI4	0.849			
	GMI5	0.882			
PI	PI1	0.849	0.892	0.921	0.699
	PI2	0.830			
	PI3	0.814			
	PI4	0.825			
	PI5	0.860			
SIR	SIR1	0.909	0.883	0.928	0.811
	SIR2	0.901			
	SIR3	0.891			
TE	TE1	0.877	0.858	0.913	0.779
	TE2	0.891			
	TE3	0.879			
TMS	TMS1	0.901	0.864	0.916	0.785
	TMS2	0.891			
	TMS3	0.865			
VR	VR1	0.870	0.897	0.928	0.764
	VR2	0.882			
	VR3	0.872			
	VR4	0.872			
VV	VV1	0.824	0.878	0.911	0.673
	VV2	0.821			
	VV3	0.767			
	VV4	0.824			
	VV5	0.861			

**Table 5:** Discriminant validity: Heterotrait–Monotrait ratio (HTMT).

	GMI	PI	SIR	TE	TMS	VR	VV
GMI							
PI	0.563						
SIR	0.492	0.361					
TE	0.481	0.430	0.324				
TMS	0.343	0.342	0.342	0.345			
VR	0.268	0.266	0.175	0.064	0.275		
VV	0.393	0.450	0.162	0.376	0.417	0.289	

**Table 6:** Discriminant validity: Fornell–Larcker criterion.

	GMI	PI	SIR	TE	TMS	VR	VV
GMI	0.867						
PI	0.510	0.836					
SIR	0.443	0.321	0.900				
TE	0.427	0.377	0.283	0.882			
TMS	0.309	0.303	0.300	0.302	0.886		
VR	0.245	0.238	0.158	0.057	0.245	0.874	
VV	0.355	0.399	0.143	0.326	0.366	0.256	0.820

**Table 7:** Results of structural model analysis.

	R^2^	R^2^ Adjusted	Q^2^ Predict
GMI	0.151	0.150	0.057
PI	0.182	0.180	0.017
TE	0.214	0.213	0.021
TMS	0.158	0.157	0.037
VR	0.025	0.024	0.022
VV	0.020	0.020	0.017

**Table 8:** Results of the path analysis test.

Hypothesis	Path	Std Beta	t	*p*-Value	95%CI	f^2^	VIF	Results
H1	SIR -> VR	0.158	4.851	<0.001	[0.095, 0.218]	0.025	1.000	Support
H2	SIR -> VV	0.143	4.315	<0.001	[0.078, 0.209]	0.021	1.000	Support
H3	VR -> GMI	0.164	5.429	<0.001	[0.104, 0.222]	0.029	1.070	Support
H4	VR -> TMS	0.161	4.955	<0.001	[0.096, 0.223]	0.028	1.070	Support
H5	VV -> GMI	0.312	10.068	<0.001	[0.250, 0.371]	0.106	1.070	Support
H6	VV -> TMS	0.325	9.966	<0.001	[0.257, 0.386]	0.115	1.070	Support
H7	GMI -> TE	0.369	11.989	<0.001	[0.308, 0.429]	0.157	1.106	Support
H8	TMS -> TE	0.188	5.770	<0.001	[0.121, 0.246]	0.039	1.106	Support
H9	TMS -> PI	0.208	5.906	<0.001	[0.137, 0.274]	0.047	1.100	Support
H10	TE -> PI	0.315	9.355	<0.001	[0.247, 0.378]	0.110	1.100	Support

**Table 9:** Necessity analysis results.

Constructs	High PI		Not High PI	
	Consistency	Coverage	Consistency	Coverage
SIR	0.738	0.686	0.591	0.644
~SIR	0.617	0.563	0.712	0.760
VR	0.708	0.723	0.552	0.661
~VR	0.668	0.561	0.769	0.755
VV	0.710	0.701	0.564	0.651
~VV	0.647	0.559	0.741	0.750
GMI	0.765	0.753	0.517	0.595
~GMI	0.589	0.510	0.785	0.796
TMS	0.721	0.711	0.567	0.654
~TMS	0.649	0.561	0.749	0.759
TE	0.728	0.705	0.567	0.643
~TE	0.632	0.555	0.740	0.761

**Table 10:** Results of configuration analysis (High PI).

Configuration	High PI								
	M1	M2	M3	M4	M5	M6	M7	M8	M9
SIR	▲	●				●			●
VR	●		●	●					●
VV		●	▲		▲		●		▲
GMI	●	●	●	●	●	●	●	●	
TMS				▲	▲			▲	▲
TE						●	●	▲	●
Consistency	0.885	0.867	0.883	0.891	0.869	0.867	0.883	0.886	0.914
Raw coverage	0.515	0.498	0.522	0.520	0.526	0.521	0.509	0.517	0.390
Unique coverage	0.008	0.004	0.007	0.003	0.004	0.020	0.013	0.004	0.014
Solution coverage	0.732								
Solution consistency	0.818								

Note: ● indicates core presence; ▲ indicates peripheral presence.

**Table 11:** Results of Configuration Analysis (Not High PI).

Configuration	Not High PI							
	N1	N2	N3	N4	N5	N6	N7	N8
SIR						✖	✖	✖
VR				✖	✖			✖
VV		✖						
GMI	✖							
TMS			✖		✖		✖	
TE			✖	✖		✖		
Consistency	0.796	0.750	0.858	0.859	0.823	0.865	0.843	0.843
Raw coverage	0.785	0.741	0.604	0.602	0.622	0.580	0.594	0.583
Unique coverage	0.031	0.018	0.003	0.005	0.006	0.005	0.001	0.004
Solution coverage	0.945							
Solution consistency	0.692							

Note: ✖ indicates core absence.

## Data Availability

The original contributions presented in this study are included in the article. Further inquiries can be directed to the corresponding author.

## Appendix A. Original Chinese Version of the Measurement Items

**Construct**	**Code**	**Original Chinese Item**
SIR	SIR1	我通常看到的线上食品图片能够提供丰富的食品感官信息。
	SIR2	这些图片能够提供充分的信息，帮助我判断食品的颜色、状态和质地等特征。
	SIR3	这些图片通常包含多种与食品味道、温度或口感有关的视觉线索。
VR	VR1	我通常看到的线上食品图片能够真实地呈现食品的外观。
	VR2	图片中食品的纹理和表面特征通常具有较强的真实感。
	VR3	图片中的食品通常具有接近真实食品的视觉效果。
	VR4	图片中的细节，如光泽、水分和表面状态，通常能够较真实地呈现食品的实际外观。
VV	VV1	观看线上食品图片时，我通常能够在脑海中形成清晰、生动的食品形象。
	VV2	线上食品图片通常能让我具体地想象出食品的外观。
	VV3	观看线上食品图片时，我通常能够形成鲜明的视觉心理表征。
	VV4	我通常能够在脑海中清晰地重现图片所呈现的食品外观细节。
	VV5	线上食品图片通常会给我留下鲜明而具体的视觉印象。
GMI	GMI1	观看线上食品图片时，我有时仿佛能够体验到食品可能具有的味道。
	GMI2	我通常能够根据线上食品图片想象食品可能具有的甜味、咸味等味觉特征。
	GMI3	观看线上食品图片时，我能够在脑海中形成较清晰的味觉感受。
	GMI4	图片中的酱料、配料等视觉信息能够使我想象食品可能具有的滋味。
	GMI5	仅通过观看线上食品图片，我通常就能够形成较为鲜明的味觉想象。
TMS	TMS1	观看线上食品图片时，我能够想象食品在口中的酥脆等质地感受。
	TMS2	我能够根据线上食品图片想象食品入口后的软硬、绵密或融化等质感。
	TMS3	我能够通过视觉信息在心理上模拟食品在口中的顺滑、绵密等质地体验。
TE	TE1	根据线上食品图片，我通常能够形成对食品是否美味的预期。
	TE2	观看线上食品图片后，我通常会对食品可能具有的味道形成判断。
	TE3	根据线上食品图片，我通常能够预期食品可能带来的口感体验。
PI	PI1	看到感兴趣的线上食品图片时，我通常会产生购买该食品的意愿。
	PI2	线上食品的视觉展示会使我对购买相关食品产生兴趣。
	PI3	看到具有吸引力的线上食品展示时，我愿意尝试相关食品。
	PI4	如果条件允许，我会考虑将感兴趣的线上食品加入购物车。
	PI5	我会考虑购买通过线上视觉展示引起我兴趣的食品。
